# Atypical Presentation of Cardiac Tamponade as a Cause of Acute Liver Injury: Case Report and Review of Literature

**DOI:** 10.7759/cureus.2779

**Published:** 2018-06-11

**Authors:** Sarang Thaker, Cemal Yazici, Sean Koppe

**Affiliations:** 1 Department of Medicine, University of Illinois at Chicago, Chicago, USA; 2 Department of Medicine, Division of Gastroenterology and Hepatology, University of Illinois at Chicago, Chicago, USA

**Keywords:** acute liver injury, cardiac tamponade, ischemic hepatitis, shock liver

## Abstract

Pericardial tamponade is a rare cause of acute liver injury due to the compressive effects of an effusion resulting in a poor cardiac output which ultimately leads to ischemia-induced injury. We present a patient with chronic hepatitis C infection and end-stage renal disease who was transferred to our center for further evaluation and management of acute liver injury after presenting to an outside hospital with left upper quadrant abdominal pain, nausea and vomiting. The patient was discovered to have tamponade physiology on transthoracic echocardiogram as an underlying cause of his acute liver injury despite lack of clinical tamponade features. He required pericardiocentesis which eventually led to resolution of the acute liver injury and he was discharged home on day twelve after full recovery. We review the existing literature regarding the epidemiology, pathophysiology, clinical presentation, diagnosis, and treatment of ischemic hepatitis, which is associated with high mortality; therefore early recognition and treatment of the underlying cause are paramount.

## Introduction and background

Ischemic hepatitis is a rare cause of acute liver injury (ALI) and is associated with various etiologies including acute cardiac failure, trauma, hemorrhage, and respiratory failure that all result in poor perfusion and oxygen delivery to the liver [1]. Patients present with elevated transaminases, often to the thousands, typically occurring after a period of hypotension; however, cases may also occur without a hypotensive episode. In fact, up to half of the cases of “shock liver” have been noted to occur in patients without shock [1]. Serum transaminases typically return to baseline level within 10 days after treating the underlying cause [2]. Patients may present with symptoms suggestive of acute hepatitis, including nausea, vomiting, abdominal pain, and malaise [3]. We present a rare case of ALI caused by pericardial tamponade in a patient with hepatitis C infection (HCV) and end-stage renal disease (ESRD) which required pericardiocentesis, ultimately leading to resolution of ALI. We also present an overview of the current literature with regards to this entity.

Case

A 56-year-old male with a past medical history of HCV, ESRD on hemodialysis, hypertension, and diabetes mellitus presented to an outside hospital with left-sided abdominal pain associated with nausea and vomiting after completion of hemodialysis earlier that day. Laboratory studies revealed elevated liver enzymes (Aspartate transaminase (AST) of 4200 U/L, alanine transaminase (ALT) of 3600 U/L, total bilirubin of 2.7 mg/dl) and coagulopathy (International normalized ratio (INR) of 2.9) along with severe lactic acidosis to 17 mmol/L. The patient was subsequently transferred to our center for evaluation and management of ALI.

Upon arrival, the patient was hypothermic to 35.7°C, but otherwise hemodynamically stable and reported resolved abdominal pain. Physical exam was remarkable for hepatomegaly and negative for distended neck veins or muffled heart sounds. Electrocardiogram (ECG) did not demonstrate low voltages.

**Figure 1:**
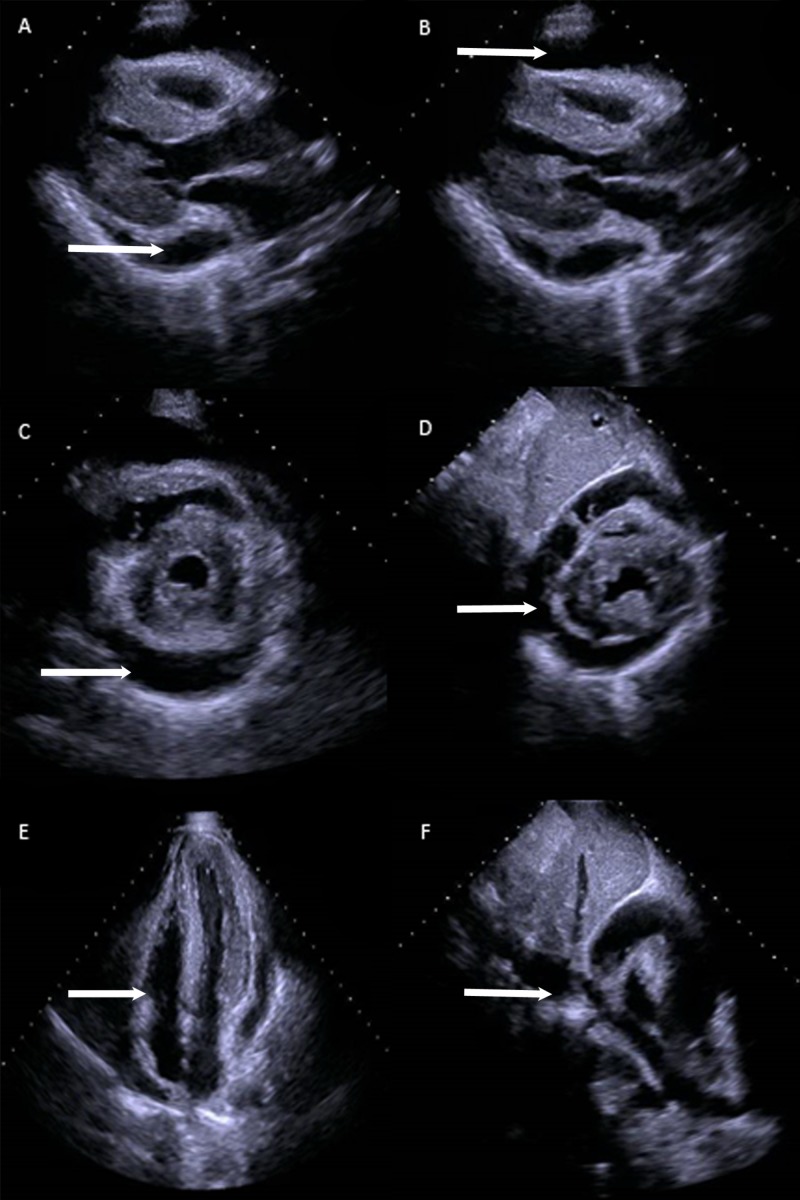
Echocardiogram. Echocardiographic findings demonstrating pericardial effusion in parasternal long view (A, B) and short axis view (C,D), flattening of interventricular septum and right ventricular collapse (E), dilated Inferior Vena Cava (F) as indicated by arrows.

**Figure 2:**
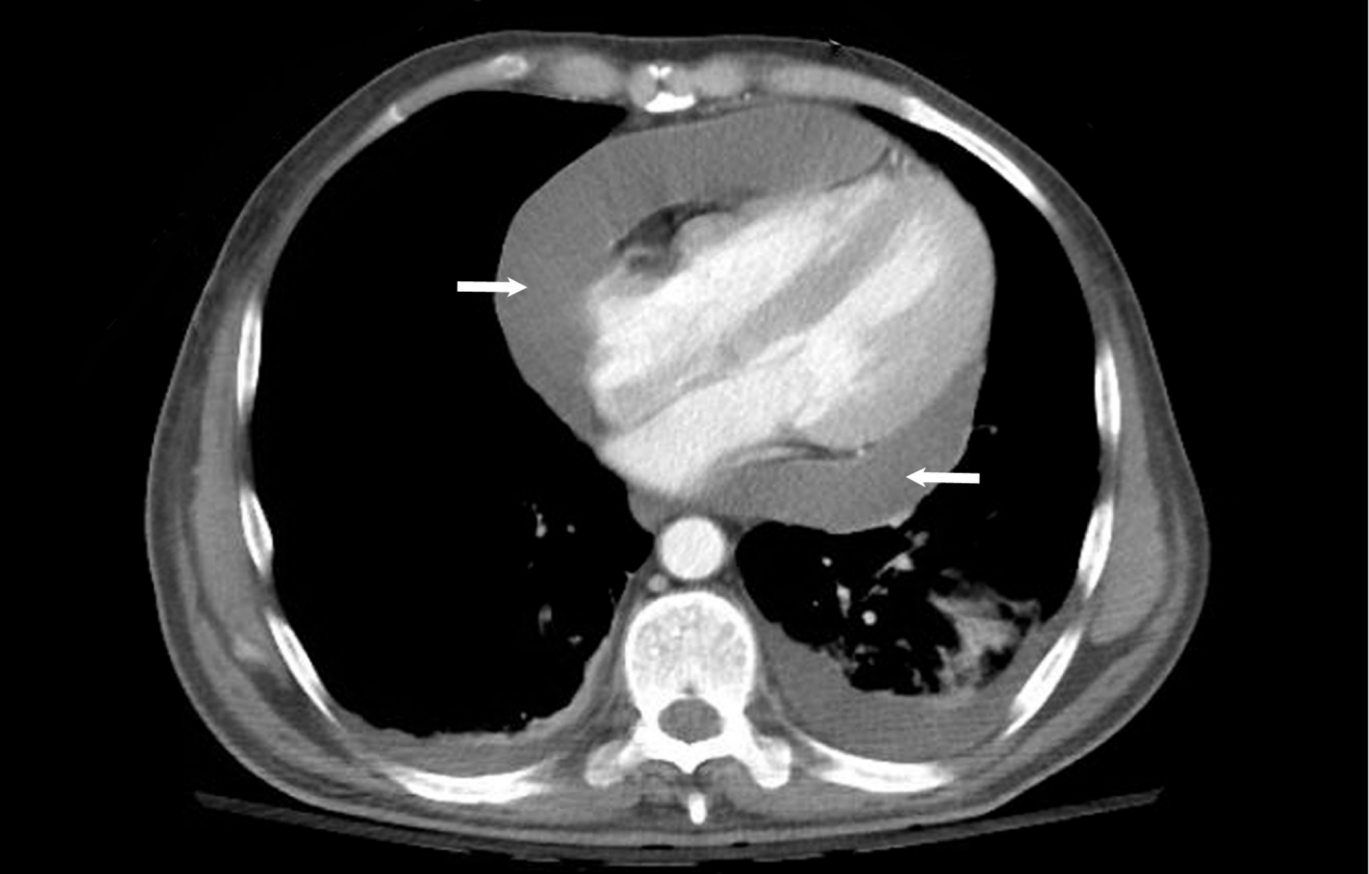
Computed tomography. Computed tomography portraying circumferential pericardial effusion (arrows).

ALI workup was initiated and abdominal ultrasonography demonstrated a normal liver with patent hepatic vasculature and a normal gallbladder without stones intrahepatic ductal dilatation, but it did note a pericardial effusion. Given these findings, an echocardiogram was performed and revealed a 2.0 cm circumferential pericardial effusion, flattening of the interventricular septum during inspiration, and a dilated inferior vena cava to 2.8 cm consistent with tamponade physiology (Figure 1) without the clinical features of tamponade. Computed tomography (CT) also revealed the presence of pericardial fluid accumulation (Figure 2). On hospital day two, the patient underwent pericardiocentesis with the removal of 500 ml of bloody fluid and drain placement. The patient improved clinically with decreasing transaminases (AST 1549 U/L and ALT 1644 U/L at 12 hours after intervention).

Later that evening, the patient’s mental status acutely worsened coinciding with hypotension and tachycardia, ultimately requiring intubation. Shortly thereafter he sustained pulseless electrical activity (PEA) arrest twice with successful return of spontaneous circulation after two and five rounds of cardiopulmonary resuscitation, respectively. Telemetry did not reveal an antecedent arrhythmia. Bedside echocardiogram did not demonstrate tamponade physiology; however, left ventricular collapse was noted indicative of volume depletion. Laboratory findings revealed a hemoglobin of 3.9 g/dL and INR of 4.9; CT head was negative for acute hemorrhage, but CT chest/abdomen/pelvis revealed hemoperitoneum and subcapsular hematoma likely secondary to pericardiocentesis. The patient was aggressively resuscitated with blood products and fluids with appropriate response and was successfully weaned off vasopressors. He was in disseminated intravascular coagulopathy (DIC) and required fresh frozen plasma, cryoprecipitate, and platelet transfusions. The patient underwent emergent right internal mammary artery embolization as it was noted that the pericardial drain coursed adjacent to this vessel. Troponinemia developed and, on post-pericardiocentesis day two, peaked at a level of 49 ng/ml with no ST elevations on electrocardiogram, deemed to be a non-ST elevation myocardial infarction (NSTEMI); however, the patient was a poor candidate for both medical and percutaneous interventions given coagulopathy and acute bleed.

**Figure 3:**
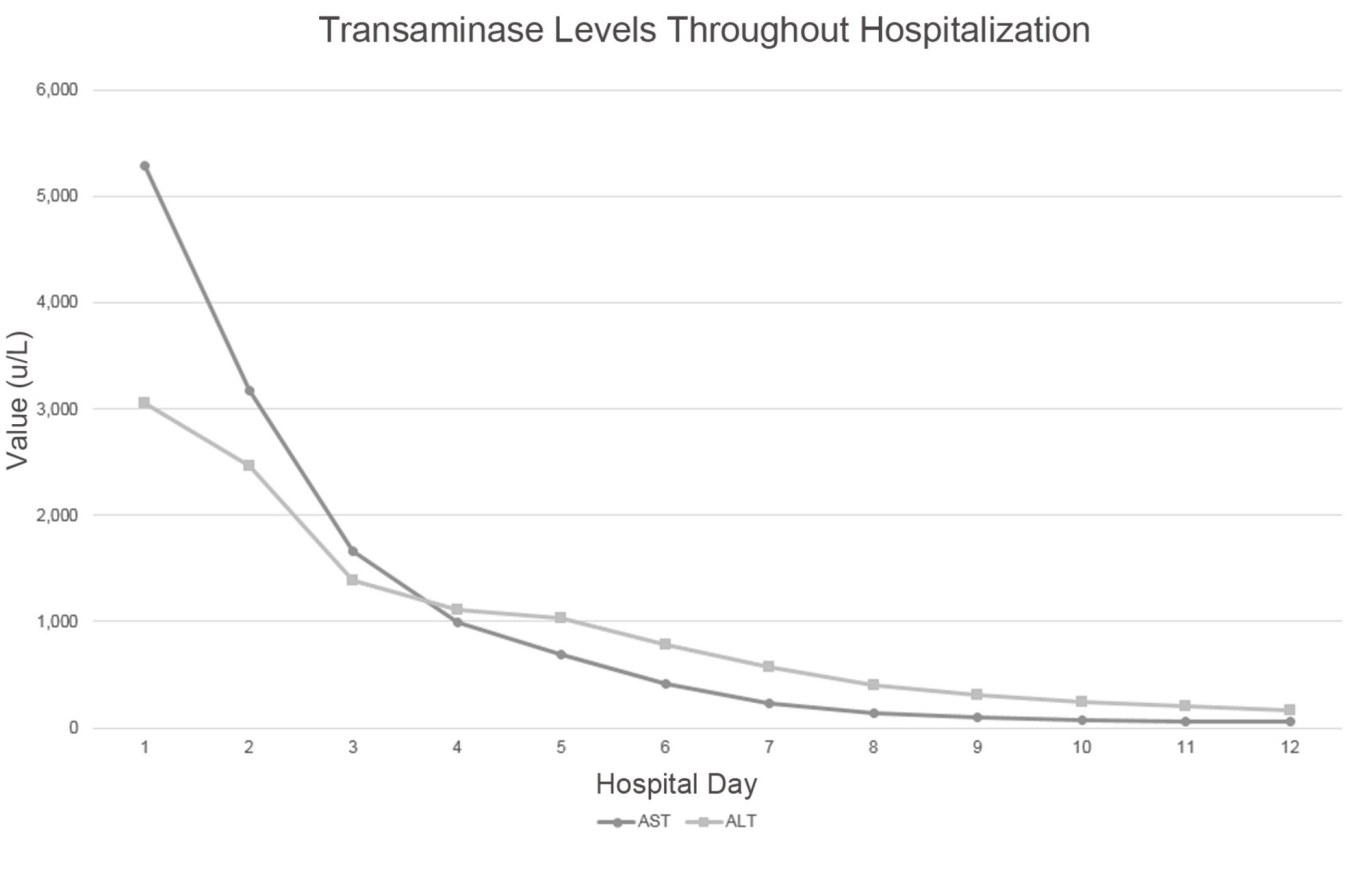
Transaminase trend. Trend of transaminases throughout hospitalization; pericardiocentesis performed on hospital day two.

Echocardiogram on hospital day four noted normal left ventricular function with regional wall motion abnormalities and reduced right ventricular function. The pericardial drain was removed, the patient was successfully extubated, DIC resolved, coagulopathy improved, and transaminases further decreased as shown in Figure 3. The patient was transferred out of the Intensive Care Unit (ICU) and the remainder of his hospital course was uncomplicated. He was safely discharged home within one week. Laboratory studies at the time of discharge were remarkable for an AST of 63 U/L, ALT of 161 U/L, INR of 1.1, and hemoglobin of 10.8 gm/dl.

The etiology of this patient’s pericardial effusion remains unclear. Cytology and anaerobic, aerobic, fungal, and acid-fast bacilli cultures were negative. Blood urea nitrogen was not significantly elevated and thus less likely the cause. Infectious workup was negative for varicella, cytomegalovirus, Epstein-Barr virus, coxsackievirus, echovirus, adenovirus, human immunodeficiency virus, tuberculosis quantiferon, histoplasmosis, and blastomycosis. Autoimmune studies including antinuclear antibody, anti-neutrophil cytoplasmic antibody, anti-mitochondrial antibody, anti-smooth muscle antibody, anticardiolipin, and anti-β2-glycoprotein were unremarkable.

## Review

Epidemiology of ischemic hepatitis

The incidence of ischemic hepatitis varies and was found to be 0.16% for general medicine ward, 0.9% for ICU and 2.6% for cardiac ICU admissions [1, 4]. The liver is generally well-protected from ischemia due to its dual blood supply from the portal vein and hepatic artery [4]. A meta-analysis including 1782 subjects from 24 studies revealed similar results [5]. Ischemic hepatitis was seen in 0.2% of all hospital admissions (both general medicine and intensive care). Ischemic hepatitis was also seen in 2.5% of ICU and 40% of all admissions in which patients had transaminase levels > 10 times the upper limit of normal. A recent single-center study noted ischemic hepatitis in 4% of ICU admissions [6].

Clinical presentation

Patients with ischemic hepatitis present with a variety of symptoms such as weakness, dyspnea, and abdominal pain. Transaminase levels increase within 48 hours after the inciting event and coagulopathy with an elevated INR subsequently occurs [4]. Bilirubin may increase as well, but jaundice is uncommon. Patients with ischemic hepatitis secondary to cardiac tamponade typically have evident signs and symptoms of tamponade on presentation. This particular patient had no evidence of cardiac compromise such as hypotension, distended neck veins, diminished cardiac sounds upon his presentation which highlights the rarity of this case. In the absence of telltale signs and symptoms of tamponade, clinicians must be suspicious of tamponade when evaluating a patient for ischemic hepatitis. Several cases of tamponade resulting in ALI have been reported, yet these patients either presented with signs and symptoms of outright tamponade or had malignancy resulting in pericardial effusion, recent cardiothoracic surgery, or *Mycobacterium tuberculosis* infection, our case had none of these [7-11]. Cardiac tamponade is a clinical diagnosis. Electrocardiogram typically demonstrates low voltages or electrical alternans which is fairly specific for tamponade; again, the patient presented above lacked these typical findings on ECG [12, 13]. Echocardiography is recommended to assess for hemodynamic compromise secondary to pericardial effusions and may reveal chamber collapse, especially right-sided, suggestive of elevated intrapericardial pressures [12]. A highly specific and sensitive finding consistent with tamponade physiology is a reversal of the normal respiratory variations of flow and volume of the ventricles in addition to a dilated inferior vena cava, which the patient in this case displayed [14, 15]. The diagnosis of tamponade in this patient was confirmed as pericardiocentesis resulted in clinical improvement as well as hemodynamic stability.

Etiology and pathophysiology

Predisposing factors include many entities, particularly cardiovascular disease, sepsis, and chronic respiratory disease. The most common cause of ischemic hepatitis was noted to be cardiac, including arrhythmias, myocardial infarction, and heart failure [1, 16]. Seeto et al. studied the pathophysiology of ischemic hepatitis in a cohort of 31 patients with ischemic hepatitis matched to 31 control patients with both groups consisting of patients with systolic blood pressures < 75 mmHg for at least 15 minutes. The group concluded that hypotension alone did not lead to ischemic hepatitis and noted that each patient that developed ischemic hepatitis had underlying cardiac disease, most commonly right-sided heart failure, which caused hepatic congestion. Indeed, as cardiac output declines or right heart pressures increase, the pressures required to perfuse the hepatic sinusoids increase resulting in reduced portal blood flow [17]. Birrer et al. and Seeto et al. concluded that transient hypotension in patients with underlying hepatic congestion due to right heart failure is sufficient to cause notable hepatocellular necrosis [18, 19]. In a recent study, Van den broecke et al. identified 1116 cases of ischemic hepatitis in a single-center ICU and determined that approximately half of these cases were as a result of cardiac failure [6]. Our patient had preexistent liver disease secondary to hepatitis C which made him relatively more vulnerable to developing ischemic hepatitis from hepatic congestion due to his pericardial effusion despite lacking overt signs of tamponade. In fact, Waseem et al. determined that patients with baseline liver disease have increased risk of developing acute liver injury in the setting of unstable hemodynamics [20]. This patient also had a history of hypertension which has been linked to diastolic dysfunction, providing another predisposing risk factor. He also has ESRD which has also been linked to the development of hepatic congestion [21]. Our patient emphasizes the role of passive hepatic congestion in the pathophysiology of ischemic hepatitis, especially when considering that his aminotransferases improved after pericardiocentesis and continued to decline even after PEA arrest and hemorrhagic shock.

In heart failure, decreased cardiac output causes a compensatory increase in systemic vascular resistance (SVR). The splanchnic vessels constrict disproportionately more than systemic vessels resulting in reduced perfusion to the liver [4]. Henrion et al. retrospectively studied the hemodynamics in 142 patients with ischemic hepatitis due to heart failure, chronic respiratory failure, and shock. This study revealed that cardiac compromise leads to liver ischemia, respiratory compromise causes hypoxemia resulting in hepatic injury, and septic and hypovolemic shock lead to an inability for the body to meet hepatic oxygenation demands [1]. Saner et al. performed a retrospective observational cohort study on 13 patients with cardiogenic shock and ALI. The group determined that patients who survived had a significantly higher cardiac index upon presentation compared to patients who did not survive, and recovery of cardiac index was associated with improved hepatic function [22]. The group noticed elevated post-shock central venous pressures (CVP) in the survival group and that hepatic function recovered despite this finding, indicating that cardiac index has more of an effect on hepatic congestion than CVP alone [22]. In a meta-analysis that included 1782 ALI cases, Tapper* *et al*. *showed that 78.2% of subjects had an inciting cardiac event, 52.9% had a hypotensive insult and 23.4% had sepsis [5].

Regarding patients with chronic respiratory failure, progressive hypoxemia causes hepatic injury, usually without an inciting event [4]. Van den broecke et al. noted that approximately 10% of ischemic hepatitis cases in a single-center ICU were related to either acute respiratory failure or acute-on-chronic respiratory failure [6]. Henrion et al. examined cases of ischemic hepatitis due to chronic respiratory failure without left-sided heart failure in order to determine if hypoxemia independently causes hepatic injury. The cohort of patients included those with restrictive and obstructive lung disease. The degree to which hypoxemia caused liver injury was confounded by systemic hypotension and elevated CVP, hence the group performed hemodynamic studies. When comparing hemodynamics between this cohort against a cohort with ischemic hepatitis due to congestive heart failure, the group revealed that the systemic hypotension in the respiratory failure group was due to reduced SVR and these patients had an increased cardiac index, whereas the heart failure group had systemic hypotension due to reduced cardiac index. When measuring oxygen delivery, the chronic respiratory failure group had profoundly low oxygen delivery due to severe hypoxemia (pO2 < 60 mmHg) [23]. The group concluded that hepatic injury in chronic respiratory failure patients is due to extreme hypoxemia primarily from progression of severe pulmonary disease and not ischemic injury due to hypotension [23]. Patients with obstructive sleep apnea or obesity hypoventilation syndrome may also develop ischemic hepatitis due to extreme hypoxemia [24]. Additionally, these patients may also have right-sided heart failure from pulmonary hypertension with subsequent hepatic congestion.

In septic shock, hepatocytes have an increased oxygen demand coupled with a reduced ability to extract oxygen resulting in injury. Van den broecke et al. noted septic shock as the cause of ischemic hepatitis in roughly 30% of ICU admissions [6]. Endotoxin stimulates Kupffer cells and causes an inflammation cascade in the parenchyma [4]. Mesenteric blood flow may be decreased due to vasoconstriction and shunting of blood elsewhere or increased due to endotoxin-mediated vasodilation. Henrion et al. noted that cardiac index was often increased due to reduced SVR, and oxygen delivery was maintained. They noted reduced intrahepatic oxygen tension indicating an increased oxygen demand and decreased extraction, resulting in inflammation and injury [1]. In fact, altered hepatic perfusion results in the release of pro-inflammatory cytokines such as tumor necrosis α and interleukin-8 resulting in dysregulated metabolism, immune response, and toxin clearance, exacerbating the underlying sepsis [25]. Activated neutrophils worsen liver injury by adhering to hepatic endothelial cells and generating reactive oxygen species [26]. Therefore, ischemic hepatitis in sepsis is primarily due to the reduced hepatic extraction of oxygen and not ischemia [1].

Other causes of ischemic hepatitis have been identified and include severe anemia where patients present with extremely low hemoglobin levels and severe lactic acidosis indicating poor tissue oxygenation. In a case report of ALI due to a hemoglobin of 1.8 g/dl, the authors postulated that anemia to this degree decreased hepatic oxygenation and resulted in acute liver injury [27]. Van den broecke et al. observed hypovolemic shock as a cause of nearly 10% of ischemic hepatitis cases in an ICU setting [6]. An additional case report noted the development of ischemic hepatitis due to septic emboli from bacterial endocarditis [28]. Cocaine-induced hepatic injury is dose-dependent and is related to P450 activity on cocaine metabolites generating oxidative stress. Cocaine also causes hepatic vasoconstrictive effect leading to hepatic injury [29]. Additionally, carbon monoxide poisoning has been shown to result in severe ischemic hepatitis due to reduced oxygen delivery to hepatocytes. In a case of carbon monoxide poisoning, resolution of the liver injury was attained with the treatment of the carboxyhemoglobinemia [30].

Diagnosis

Ischemic hepatitis is typically diagnosed by laboratory studies demonstrating an acute hypertransaminasemia, usually > 100 times the upper limit of normal [31]. Differential diagnosis includes viral and autoimmune hepatitis, direct liver trauma, vascular disease, toxin-induced such as acetaminophen ingestion, and biliary obstruction secondary to lithiasis. Liver biopsy is rarely performed and usually reveals centrilobular necrosis [4, 7]. Pathological changes related to ischemic hepatitis are usually observed in zone III near the central vein [4]. The first step in the evaluation of elevated aminotransferases is to obtain abdominal ultrasonography to exclude choledocholithiasis especially in patients with elevated bilirubin, abdominal pain, and signs concerning for infection such as our patient. Next, the pattern of aminotransferase can indicate the etiology. Our patient had an AST > ALT which would normally suggest alcohol ingestion, however the degree of elevation seen in ischemic hepatitis is much greater than that of alcoholic hepatitis which does not exceed > 10 times the upper limit of normal, substantiating the diagnosis of ischemic hepatitis especially in setting of a normal ultrasound [32]. The case above highlights the complexity of ischemic hepatitis etiologies and emphasizes the necessity of clinical suspicion and obtaining prompt echocardiography in patients with an otherwise unremarkable evaluation.

Prognosis and treatment

In a retrospective study of 142 ICU patients, 30-day mortality rate of ischemic hepatitis was 52.8% and one-year survival rate was 28.3% with the caveat that this patient population either had decompensated heart failure, exacerbation of chronic respiratory failure, or circulatory shock [1]. Birrer et al. noted a mortality rate of 45% in 293 cases and death occurred due to sudden cardiorespiratory failure [19]. In a meta-analysis with 1782 cases, the mortality rate of ischemic hepatitis was noted to be approximately 50% [5]. In a single-center cohort of 1116 ICU patients with ischemic hepatitis, Van den broecke et al. observed a 45.0% mortality rate at 28 days with 18.5% of cases dying within 24 hours of peak AST level [6]. This study also differentiated mortality rates based on etiology of ischemic hepatitis. The septic shock subgroup and cardiac failure subgroup had a 28-day mortality rate of 52.9% and 41.4%, respectively [6]. Fuhrmann et al. showed a statistically significant higher peak of aminotransferases, INR, and lactate was observed in patients who died [33]. Clearly, there is a significant mortality risk in ischemic hepatitis and early identification of underlying etiology and its timely treatment is key for survival as demonstrated by this case.

Treatment of ischemic hepatitis is targeted towards correcting the underlying etiology. In patients who develop ALI secondary to cardiac tamponade, pericardiocentesis should be performed and the resultant resolution of transaminitis is typically seen within 72 hours. Additionally, correction of pathology that leads to the formation of pericardial effusion is recommended. Elevated liver enzymes in patients with ischemic hepatitis typically undergo a 50% reduction within 72 hours of correcting the underlying cause [4].

Patients with heart failure and elevated CVP may require inotropic-supported diuresis to decrease hepatic congestion and increase splanchnic blood flow [5]. Septic shock patients benefit from vasopressors, antimicrobials, fluid resuscitation, and supportive care [5]. Hepatobiliary transport is reduced in sepsis leading to cholestasis. Resultant translocation of endotoxin and endotoxin-mediated response further worsens sepsis as detailed earlier [26]. Antimicrobial selection should aim to treat intra-abdominal organisms such as Gram-negative rods and anaerobes in septic patients with findings and clinical signs of intra-abdominal source.

In patients with chronic respiratory failure, treatment should be directed towards correcting hypoxemia as well as treating the underlying cause with supplemental oxygen, possible ventilatory support, nebulized beta-agonists and anticholinergics, corticosteroids, and possibly antimicrobials [34]. As noted above, both restrictive and obstructive lung disease patients can develop ischemic hepatitis; therefore, the etiology of chronic respiratory failure should be managed with its respective treatment [23]. Patients with obstructive sleep apnea should be treated with non-invasive positive pressure ventilation to minimize nocturnal oxygen desaturation [24]. Finally, in patients with right-sided heart failure due to pulmonary hypertension, treatment must emphasize improving hemodynamics and addressing the etiology of pulmonary hypertension [35].

Ischemic hepatitis due to toxins such as cocaine and 3,4-methylenedioxymethamphetamine (MDMA) ingestion is largely treated with supportive care as they present with a constellation of symptoms including, but not limited to, hyperthermia, rhabdomyolysis, hypotension, and DIC [29]. As discussed earlier, roughly one-half of patients with acute liver injury due to ischemic hepatitis do not survive to discharge, therefore it is paramount that early recognition and diagnosis be made in order to expedite treatment [19].

## Conclusions

This case emphasizes the requirement for proper workup of any patient presenting with the acute liver injury. As various studies have explained, the most common causes of elevated aminotransferases to the thousands include toxins, trauma, acute viral hepatitis, and ischemic hepatitis which is most often cardiac in origin due to arrhythmia, myocardial infarction, heart failure, or tamponade as in this patient. Though this patient did not exhibit the classic signs and symptoms of pericardial tamponade, it nonetheless was the cause of the presenting ALI. Furthermore, as the review of the literature has demonstrated, ischemic hepatitis is prevalent and associated with high mortality, underscoring the necessity for vigilance and early recognition in order to improve outcomes.
